# Enterocolic Lymphocytic Phlebitis Masquerading as a Malignant Stricture in the Caecum: A Case Report and Literature Review

**DOI:** 10.7759/cureus.72699

**Published:** 2024-10-30

**Authors:** Dakshita Agrawal, Rakshita Agrawal, Sangara Narayanasamy, Sadhasivam Ramasamy, Ali Yasen Mohamedahmed, Pradeep Thomas, Najam Husain

**Affiliations:** 1 General Surgery, Queen's Hospital Burton, University Hospitals of Derby and Burton NHS Foundation Trust, Burton-on-Trent, GBR

**Keywords:** abdominal malignancy, caecal cancer, enterocolic lymphocytic phlebitis, histological analysis, venous inflammation

## Abstract

Enterocolic lymphocytic phlebitis (ELP) is an idiopathic form of venous inflammation that is limited to the gastrointestinal tract. It is characterised by inflammation of the veins with no involvement of the arteries. Usually, it presents with gastrointestinal symptoms such as abdominal pain, and imaging may suggest malignancy. The reported case is a 56-year-old female presented with rectal bleeding, abdominal pain and diarrhoea. All blood test results were normal; however, she had an elevated C-reactive protein (CRP) level. Computed tomography colonography showed caecal wall thickening, suggesting caecal malignancy. Following a multidisciplinary discussion, the patient had a right hemicolectomy, and the histology showed no malignancy and typical features of ELP. This is a rare idiopathic form of venous inflammation localised to the gastrointestinal tract, which can occasionally present similarly to bowel malignancy. ELP most commonly presents with abdominal pain, followed by hematochezia and diarrhaea. The duration of symptoms varies widely, from hours to a year. Diagnosis is usually confirmed by histopathological assessment. We present this case and literature review considering its rarity, which adds to the literature on this condition.

## Introduction

Enterocolic lymphocytic phlebitis (ELP) is inflammation of the veins within the gastrointestinal tract that mainly affects the bowel wall and commonly presents with features indicative of bowel obstruction [1]. Despite ELP presenting as an uncommon cause of bowel obstruction, the aetiology and pathophysiology of ELP remain unclear. Notably, this condition is differentiated by the exclusive involvement of veins, with no associated arterial manifestations or indicators of systemic vasculitis. 

Given the rarity of ELP, diagnosis is challenging as it presents similar to the common gastrointestinal conditions, leading to misdiagnosis or delayed diagnosis [2]. The potential complications of ELP include bowel ischemia and the risk of misdiagnosis as malignancy or other inflammatory bowel diseases, highlighting the importance of understanding this condition. Histopathological examination of the resected tissue is essential to confirm the diagnosis, revealing the characteristic lymphocytic infiltration of the venous walls [3]. However, there is no consensus on the treatment protocol for ELP; understanding the characteristic features of ELP is crucial for appropriate surgical management [2]. 

This case report details a rare instance of ELP presenting with features mimicking cancer of the caecum, along with a review of similar cases. ELP is considered a rare condition, with only a limited number of cases documented in the medical literature. Its symptoms often mimic those of more common gastrointestinal disorders, and this highlights the need for greater awareness and further research into ELP and its management.

## Case presentation

**Figure 1:**
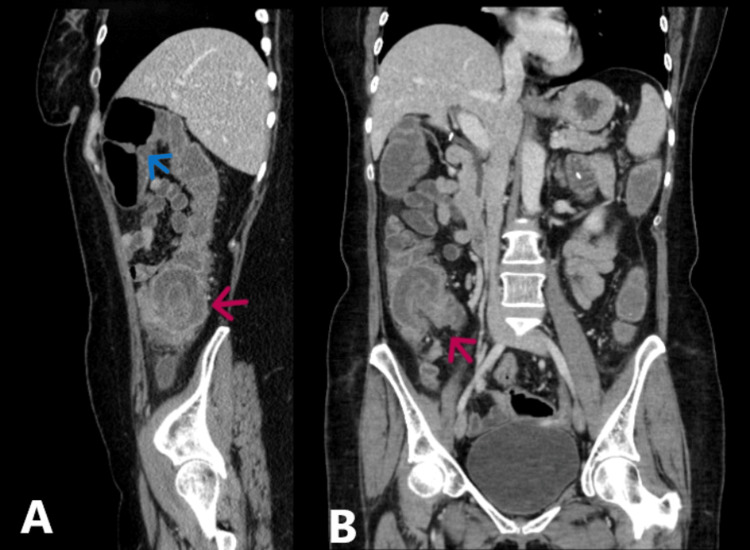
CT abdomen and pelvis demonstrating circumferential thickening within the mid-transverse colon (blue arrow) and features of telescoping/ileocaecal intussusception of the terminal ileum onto the caecum (red arrows) CT: Computed tomography

A 56-year-old female presented to the hospital with six episodes of fresh red blood in her stools, abdominal pain, and diarrhoea for one month. The patient has a past medical history of gastroparesis, postural tachycardia syndrome, type 2 diabetes mellitus, and fibromyalgia. Blood test results were normal. Rectal examination revealed the presence of haemorrhoids. Consequently, she was treated conservatively for haemorrhoidal bleeding with outpatient follow-up. Two days later, the patient presented to the emergency department with worsening central abdominal pain, diarrhoea, episodes of pyrexia, and one episode of vomiting since the previous visit. On examination, there was right iliac fossa tenderness, and blood analysis was performed. Full-blood count and urea and electrolytes were all within normal range apart from a raised C-reactive protein (CRP) and mildly raised neutrophil count (Table 1). Computed tomography (CT) of the abdomen and pelvis showed circumferential thickening within the mid-transverse colon, which was concerning for colonic cancer (blue arrow, Figure 1). Moreover, there were features of intussusception of the terminal ileum into the caecum at the ileocaecal junction (red arrows, Figure 1).

**Table 1:** Blood test analysis. Abnormal CRP and neutrophil count L: Litre; g: gram; mmol: millimoles; ml: millilitre; min: minute; fL: femtolitre; MCV: mean corpuscular volume; CRP: C-reactive protein; eGFR: estimated glomerular filtration rate

Blood test	Result	Units	Range
White cell count	10.0	10*9/L	4.0-10.0
Neutrophils	7.5	10*9/L	2.0-7.0
Lymphocytes	1.4	10*9/L	1.0-3.0
Haemoglobin	130	g/L	120-150
MCV	87.7	fL	83.0-101.0
CRP	105.0	mg/L	0.0-5.0
Sodium	134.0	mmol/L	133.0-146.0
Potassium	3.7	mmol/L	3.5-5.3
Urea	2.5	mmol/L	2.5-7.8
Creatinine	57.0	micromol/L	45.0-84.0
eGFR	99.9	ml/min	90.0-120.0

**Figure 2:**
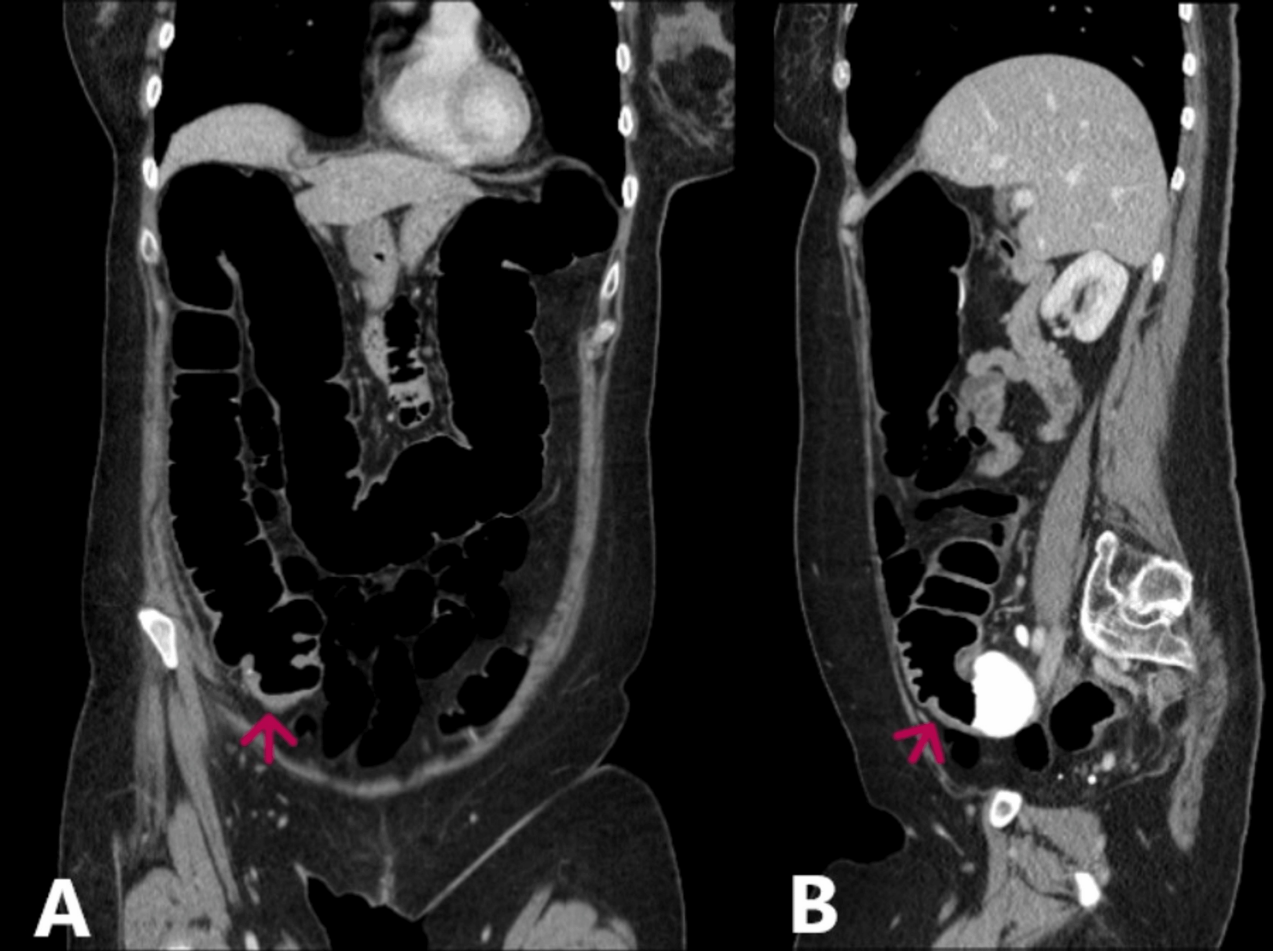
CT colongram shows moderately tortuous colon with suspicious caecal wall thickening (red arrows) CT: Computed tomography

**Figure 3:**
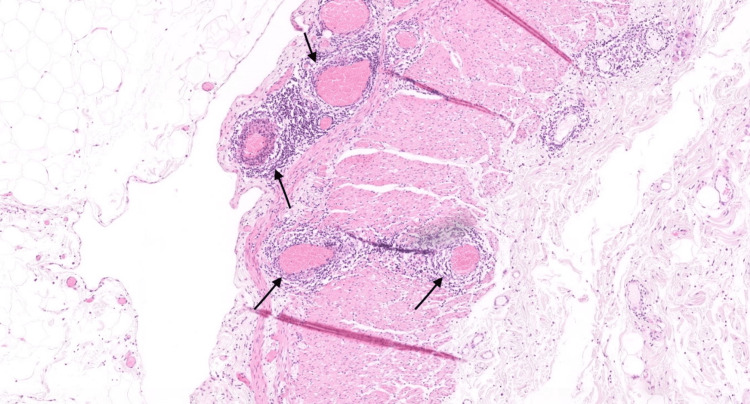
Histology findings. Histological analysis of the specimen showing lymphocytic infiltration in the veins (black arrows) with no arterial involvement

The patient was discharged the following day with a short course of oral antibiotics and urgent outpatient colonoscopy, which was incomplete due to an unresolvable sigmoid loop. CT colonography showed a moderately tortuous colon with suspicious wall thickening in the caecum opposite the ileocaecal junction (Figure 2). Subsequently, she underwent right hemicolectomy following the colorectal multidisciplinary meeting recommendations in December 2023. Histopathological examination of the specimen revealed microscopic changes of chronic ischemia on the background of widespread inflammation of the veins of the ileum, colonic wall, and mesentery. Moreover, there were no macroscopic changes, as no perforations, polyps, or masses were identified. These findings confirmed the diagnosis of ELP of the submucosa, subserosa, and mesenteric veins. The veins showed lymphocytic infiltration of the wall with the formation of lymphocytic cuffs surrounding the veins; the arteries were unaffected, and no granulomas, dysplasia, or malignancy were observed (Figure 3). The patient is currently under surgical follow-up and has not been discharged. 

## Discussion

Literature review 

Methods

A comprehensive search was conducted across various online databases such as PubMed, MEDLINE, and Google Scholar. The primary term used in the literature search was ‘enterocolic lymphocytic phlebitis'; additional terms such as ‘mesenteric phlebitis’, ‘phlebitis’, ‘abdominal pathology’, and ‘histological diagnoses’ were also utilised. The inclusion criteria of the literature review were case reports with a diagnosis of ELP, and there were no restrictions on the age and sex of the patients, the publication year of the articles, or the geographical location of the case reports for this review. 

Cases which mentioned or discussed about other conditions such as mesenteric inflammatory veno-occlusive disease (MIVOD) and idiopathic myointimal hyperplasia of the mesenteric veins (IMHMV) were excluded. MIVOD and IMHMV are other types of bowel ischaemia in which the veins are affected and were excluded because of the ambiguous and overlapping nature of these conditions with ELP. Therefore, only articles specifically addressing ELP were included. Once potentially relevant articles were identified based on their titles or abstracts, they were further evaluated for inclusion in the review. Additional cases were identified from the reference lists of the eligible articles, and if they discussed ELP, they were selected for literature review.

Results 

A total of 29 cases were identified from the literature review (Table 1) comprising 55.2% male and 44.8% female patients, with ages ranging from nine and 81 years old. Symptom duration varied widely, from hours to as long as one year, though symptoms typically persisted for hours to a few days before the patient presented to the hospital. The majority of cases (96.6%) reported ELP being in the lower gastrointestinal tract with only one case reporting ELP presence in the upper gastrointestinal tract and only 3.45% of cases reported a recurrence of ELP. 

As histological analysis is the definitive method for diagnosis, only 93.1% (27 cases) included histological findings, all of which revealed mucosal changes. In addition to this, 55.2% (16 cases) reported the size of the vein being affected, but 100% of these 16 cases showed that ELP only affects small to medium mesenteric veins. A total of 28 cases mentioned about arterial involvement, but 100% of these 28 cases showed no arterial involvement, and 93.1% of these cases explained lymphocytic involvement in histology findings.

Patients presented with a variety of symptoms, with abdominal pain being the most prevalent (86.2%), followed by haematochezia (34.5%), diarrhoea (24.1%), and fever (13.8%). Additionally, nausea, vomiting, and presence of abdominal mass were each reported by 10.3% of patients. A total of 6.9% of patients were either asymptomatic or presented with acute abdomen, and 3.45% of the cases reported fatigue, dyspepsia, melena, and haematemesis. Table 2 presents the characteristics of the cases included in the literature review.

**Table 2:** Attributes of case reports. Twenty-nine cases identified within the literature with case reports published regarding ELP ELP: Enterocolic lymphocytic phlebitis; NA: not available; M: male; F: female

Author and year	Age and sex	Symptoms	Duration of symptoms	Histology and cell classification	Size of vein affected
Abraham et al., 2004 [4]	68M	Abdominal pain, melena, and haematemesis	9 months	Submucosa, muscularis propria with lymphocytes, giant cells, histiocytes, plasma cells, and eosinophils	Small
Arain et al., 2002 [5]	65M	Nausea, vomiting, diarrhoea, abdominal pain, haematochezia, and abdominal mass	2 weeks	Serosa and mesentery with lymphocytes and eosinophils	NA
Arora et al., 1999 [6]	79M	Abdominal pain, dyspepsia, and haematochezia	15 months	Submucosa and subserosa with lymphocytes	NA
Bowee et al., 2023 [2]	81M	Lower abdominal pain and fever	11 days	Submucosa with admixed eosinophils, plasma cells, and lymphocytes	Small to medium
Chan and Toon, 2021 [7]	48F	Abdominal pain	NA	Submucosa with lymphocytes and occasional eosinophils	NA
Farber et al., 2021 [3]	65M	Abdominal pain, diarrhoea, and haematochezia	6 months	Submucosa with lymphocytes	NA
Galazka et al., 2012 [8]	35F	Abdominal pain, vomiting, and diarrhoea	2 days	Submucosa, subserosa with lymphocytes, histiocytes, and giant multinucleated cells	Small
Ghersin et al., 2013 [9]	37M	Abdominal pain	Hours	Submucosa and subserosa	NA
Huiberts et al., 2014 [10]	42F	Abdominal pain, abdominal mass, and nausea	NA	Lymphocytes	NA
Jain and Chetty, 2009 [11]	73M	Asymptomatic (iron-deficiency anaemia)	NA	Submucosa with lymphocytes and eosinophiles	NA
Laco et al., 2015 [12]	65F	Asymptomatic (anaemia)	NA	Submucosa with lymphocytes and plasma cells	Small
Massasso et al., 2007 [13]	54F	Abdominal pain	2 days	Submucosa with lymphocytes	Small
	50M	Abdominal pain	Hours	Submucosa with lymphocytes	Medium
Medlicott et al., 2006 [14]	37M	Abdominal pain, fever, nausea, and vomiting	16 hours	Submucosa, subserosa with lymphocytes, histiocytes, and neutrophils	Small
Mora-Guzman and Alonso-Casado, 2019 [15]	60F	Abdominal pain, watery diarrhoea, and haematochezia	Acute onset	Submucosa with lymphocytes	NA
Nallamothu et al., 2011 [16]	29M	Abdominal pain, haematochezia	2 days	Submucosa with lymphocytes	NA
Nasher and Alizai, 2021 [17]	9F	Abdominal pain	2 days	NA	NA
Okano et al., 2022 [18]	47M	Abdominal pain	1 year	Submucosa with lymphocytes	Small to medium
Pares et al., 2003 [19]	50F	Haematochezia	8 hours	Submucosa with lymphocytes	NA
Saraga and Costa, 1989 [1]	58M	Abdominal pain, haematochezia, fever	2 weeks	Submucosa and mucosa with lymphocytes and plasma cells	Small to medium
	77F	Abdominal pain	10 days	Submucosa and mucosa with lymphocytes and plasma cells	Small to medium
	66M	Abdominal pain and haematochezia	2 days	Submucosa and mucosa with lymphocytes and plasma cells	Small to medium
Saraga and Bouzourenne, 2000 [20]	67M	Abdominal pain and acute abdomen	8 hours	Submucosa and mucosa with neutrophiles and lymphocytes	Small to medium
	70F	Abdominal pain and fever	1 week	Submucosa and mucosa with lymphocytes	Small to medium
	72F	Acute abdomen	12 hours	Submucosa and mucosa with lymphocytes and neutrophiles	Small to medium
Seo et al., 2014 [21]	38F	Abdominal pain, haematochezia, and diarrhoea	36 hours	Submucosa and subserosa with lymphocytes	Small
Shiraki et al., 2004 [22]	73M	Abdominal pain, diarrhoea, fatigue, and mass	3 weeks	Submucosa and muscularis propria with lymphocytes	Small to medium
Tuppy et al., 2000 [23]	74F	Abdominal pain	NA	Submucosa and subserosa with lymphocytes	NA
Wright and Cacala, 2004 [24]	53M	Abdominal pain, haematochezia, and diarrhoea	12 hours	Submucosa, subserosa, muscularis propria with lymphocytes, plasma cells, eosinophils, and histiocytes	NA

Discussion

The clinical manifestations of ELP vary considerably. Symptoms typically include abdominal pain (mild or intense), multiple episodes of diarrhoea, and haematochezia, along with nausea and vomiting. These symptoms can have a more chronic onset; however, patients often present with acute abdomen [2,3]. In this review, four patients presented with an abdominal mass resembling a carcinoma during clinical examination [4-6]. Additionally, ileocecal intussusception was identified intraoperatively, most frequently in the caecum [2,6-14]. However, there have been cases of ELP in which patients have presented with no specific abdominal symptoms [9,10].

Although no definitive imaging findings are associated with ELP, bowel wall thickening or colonic masses are commonly identified [3]. The diagnosis of ELP is confirmed by histopathological assessment, and surgical resection of the affected bowel typically offers a curative outcome without recurrence [15]. Recurrence is rare and has been reported in only one case [16]. Moreover, there have been no reported instances of systemic vasculitis resulting from ELP [17]. There are no established risk factors for ELP; however, associations have been noted between ELP and the use of two different medications. In 1989, Saraga et al. reported three cases of intestinal ischaemia due to ELP, which they attributed to the use of hydroxyethylrutoside, a medication commonly used for varicose vein treatment in Europe [1]. Furthermore, ELP development has been associated with the use of anti-androgen drugs such as flutamide [18,19].

Lesions associated with ELP are primarily observed in the lower gastrointestinal tract. Specifically, ELP tends to occur mainly in the right colon of the large bowel. Furthermore, the involvement of the appendix has been reported [15], with a recent case of a nine-year-old child with hyperplasia in the appendix wall during surgical exploration [20]. In addition to this, Abraham et al. reported a case of ELP in the stomach which presented with features of peptic ulcer caused by vasculitis [4]. One of the principal histological features of ELP is that the arteries remain unaffected, whereas the veins exhibit notable lymphocytic inflammation. Most commonly, small-to medium-sized veins are affected by this disease [21]. These affected vessels are frequently situated within the mucosa and submucosa of the bowel wall [16]. 

Histological evaluation of ELP reveals a wide range of abnormalities, such as lymphocytic involvement of the venular wall, necrotising phlebitis, granulomatous phlebitis, and endothelial and myointimal hyperplasia [15]. In addition, histological analysis frequently reveals the existence of venous thrombi. Venous inflammation precipitates the formation of these thrombi, subsequently resulting in tissue oedema and compression, and finally necrosis and consequent intestinal ischaemia [16,19]. 

Lymphocytic phlebitis manifests as the infiltration of lymphocytes throughout all layers of the vein wall, in contrast to necrotising phlebitis, which is characterised by neutrophils within the veins surrounded by lymphocytic cells along the periphery [17]. Granulomatous phlebitis occurs when epithelioid and giant cells are centrally located and accompanied by lymphocytic cells at the periphery. Myointimal hyperplasia results from vein lumen occlusion. Flaherty et al. proposed a sequential progression in the stages of ELP, starting with necrotising phlebitis, followed by lymphocytic phlebitis, and concluding with myointimal hyperplasia [25]. Myointimal hyperplasia has been reported as the postoperative histological findings in several other case reports [7,16,23,26]. Immunostaining of the affected veins predominantly revealed many cluster of differentiation (CD) T cells such as CD3+, CD4+, and CD8+ T cells, whereas, CD20+ B cells were located in the surrounding regions. Other cells, such as activated natural killer cells and cytotoxic T cells, exhibit a nonspecific distribution pattern [27].

In addition, histological analysis of one resected specimen revealed submucosal haemorrhage and polypoid granulation tissue [7], while analysis of a specimen following subtotal colectomy in another case showed trans-mural ischaemic lesions with necrotic mucosa [15]. These cases highlight the significant variability in histological findings across different instances of ELP, emphasising the need for careful consideration in histological evaluations. Notably, both cases demonstrated lymphocyte infiltration in the venous walls, providing key diagnostic support for ELP [7,15].

ELP typically occur independently of other types of colitis. However, two case reports have documented a link between lymphocytic phlebitis and colitis. In the first case report, Arora et al. documented a case where lymphocytic phlebitis was present proximally, whereas lymphocytic colitis was present distally [6]. Similarly, Wright and Cacala described a case of ELP resembling lymphocytic colitis, lymphocytic enteritis, and lymphocytic appendicitis, characterised by high amounts of intraepithelial lymphocytes. Notably, T lymphocytes constitute the predominant inflammatory cell type in both ELP and lymphocytic colitis, suggesting a potential link between these two disorders [24]. However, in both cases, the patients were exposed to flutamide, suggesting that it might have triggered the condition [6,24]. 

In a documented case, the patient initially presented with symptoms of abdominal pain, diarrhoea, and a mass. Diagnostic procedures initially suggested panniculitis of the descending colon; however, histological examination of the mass revealed ELP due to the presence of lymphocytes in the veins. Furthermore, the presence of inflammatory cells in the fat tissue of the colonic mesentery indicated that ELP might have caused or contributed to the development of panniculitis in this particular case. This suggests that ELP could potentially be a cause or trigger of panniculitis in some instances [5].

Surgical resection is considered the primary approach for treating ELP, as it effectively removes the affected tissue and minimises the risk of recurrence. This approach directly address the underlying issue aiming to prevent further complications. After the surgery, patients typically experience significant clinical improvement as the source of the problem has been resolved. Recovery and long-term outcomes often depend on the extent of the diseased patients' overall health, but surgery generally leads to positive results [2].

## Conclusions

ELP remains a rare condition, with limited available information. Clinically, it presents with abdominal pain, a palpable mass, and diarrhoea. Imaging often lacks specific features indicative of ELP and commonly shows bowel thickening and colonic mass. Surgical intervention is the preferred diagnostic and therapeutic strategy. Histological examination consistently revealed lymphocytic infiltration confined to the veins while sparing the arteries. Further investigations and research are required to advance our understanding of ELP and its underlying pathogenesis, thus aiding diagnostic and therapeutic approaches and recognising the association between ELP and other colon pathologies.

## References

[1] Saraga EP, Costa J (1989). Idiopathic entero-colic lymphocytic phlebitis: a cause of ischemic intestinal necrosis. Am J Surg Pathol.

[2] Bowee S, Matter SB, Dawson H, Inglin RA (2023). Enterocolic phlebitis: a rare cause of bowel ischemia and review of the literature. Gastroenterol Rep (Oxf).

[3] Farber ON, Weingarden A, Lee C (2021). Not in the same vein: inflammatory bowel disease, malignancy, and enterocolic lymphocytic phlebitis. Dig Dis Sci.

[4] Abraham SC, Solem CA, Hauser SC, Smyrk TC (2004). Chronic antral ulcer associated with gastroduodenal lymphocytic phlebitis. Am J Surg Pathol.

[5] Arain FA, Willey J, Richter J, Senagore A, Petras R (2002). An unusual presentation of enterocolic lymphocytic phlebitis. J Clin Gastroenterol.

[6] Arora DS, Mahmood T, Wyatt JI (1999). Lymphocytic venulitis: an unusual association with microscopic colitis. J Clin Pathol.

[7] Chan N, Toon C (2021). Enterocolic lymphocytic phlebitis presenting as a caecal mass. Histopathology.

[8] Gałązka K, Tokarek T, Gach T, Szpor J (2012). Enterocolic lymphocytic phlebitis: an unusual cause of abdominal complaints. Pol J Pathol.

[9] Ghersin I, Sabo E, Lachter J (2013). Enterocolic lymphocytic phlebitis preceding the development of inflammatory bowel disease: report of a case. Rev Esp Enferm Dig.

[10] Huiberts AA, Donkervoort SC, Blok WL, Blaauwgeers HL (2014). Enterocolic lymphocytic phlebitis: an oncologic surgical resection without a preoperative pathologic diagnosis. J Surg Case Rep.

[11] Jain R, Chetty R (2009). Enterocolic lymphocytic phlebitis and lymphocytic colitis: drug-related coexistent pathology. Int J Colorectal Dis.

[12] Laco J, Örhalmi J, Bártová J, Zimandlová D (2015). Enterocolic lymphocytic phlebitis as a newly recognized manifestation of IgG4-related disease. Int J Surg Pathol.

[13] Massasso D, Henderson C, Davies D, Sugrue M, Broadfoot A, Joshua F (2007). Enterocolic lymphocytic phlebitis: a rare mimic of appendicitis and bowel cancer. ANZ J Surg.

[14] Medlicott SA, Guggisberg KA, DesCôteaux JG, Beck P (2006). Enterocolic lymphocytic phlebitis: statistical analysis of histology features in viable and ischemic bowel. Int J Surg Pathol.

[15] Mora-Guzmán I, Alonso-Casado AP (2019). Enterocolic lymphocytic phlebitis: a mimicking entity. Int J Surg Pathol.

[16] Nallamothu G, Hilden K, Karnam U, Adler DG (2011). Enterocolic lymphocytic phlebitis presenting as an intussuscepting cecal mass. Am J Gastroenterol.

[17] Nasher O, Alizai N (2021). Enterocolic lymphocytic phlebitis: a rare pathology in children. BMJ Case Rep.

[18] Okano S, Yao T, Nomura O (2022). Enterocolic lymphocytic phlebitis treated preoperatively with biologics and immunosuppressive agents. Case Rep Pathol.

[19] Parés D, Biondo S, Martí-Ragué J, Vidal A, Kreisler E, Jaurrieta E (2003). Enterocolic lymphocytic phlebitis of the right colon as a cause of massive gastrointestinal bleeding. Colorectal Dis.

[20] Saraga E, Bouzourenne H (2000). Enterocolic (lymphocytic) phlebitis: a rare cause of intestinal ischemic necrosis: a series of six patients and review of the literature. Am J Surg Pathol.

[21] Seo MR, Kim TE, Ryu HJ, Baek HJ, Choi HJ (2014). A case of enterocolic lymphocytic phlebitis mimicking surgical abdomen. J Rheum Dis.

[22] Shiraki M, Takagi S, Watanabe M (2004). Panniculitis of the descending colon caused by enterocolic phlebitis: a case report. Tohoku J Exp Med.

[23] Tuppy H, Haidenthaler A, Schandalik R, Oberhuber G (2000). Idiopathic enterocolic lymphocytic phlebitis: a rare cause of ischemic colitis. Mod Pathol.

[24] Wright CL, Cacala S (2004). Enterocolic lymphocytic phlebitis with lymphocytic colitis, lymphocytic appendicitis, and lymphocytic enteritis. Am J Surg Pathol.

[25] Flaherty MJ, Lie JT, Haggitt RC (1994). A seldom recognized cause of intestinal ischemia. Am J Surg Pathol.

[26] Nakaya M, Hashimoto H, Nagata R (2019). Enterocolic lymphocytic phlebitis with marked myointimal hyperplasia and perivenous concentric fibrosis. Cardiovasc Pathol.

[27] Ngo N, Chang F (2007). Enterocolic lymphocytic phlebitis: clinicopathologic features and review of the literature. Arch Pathol Lab Med.

